# Dual Role of Autophagy in Regulation of Mesenchymal Stem Cell Senescence

**DOI:** 10.3389/fcell.2020.00276

**Published:** 2020-04-24

**Authors:** Raffaella Rastaldo, Emanuela Vitale, Claudia Giachino

**Affiliations:** Department of Clinical and Biological Sciences, University of Turin, Turin, Italy

**Keywords:** mesenchymal stem cell, senescence, general autophagy, selective autophagy, SASP

## Abstract

During their development and overall life, mesenchymal stem cells (MSCs) encounter a plethora of internal and external stress signals and therefore, they need to put in action homeostatic changes in order to face these stresses. To this aim, similar to other mammalian cells, MSCs are endowed with two crucial biological responses, autophagy and senescence. Sharing of a number of stimuli like shrinkage of telomeres, oncogenic and oxidative stress, and DNA damage, suggest an intriguingly close relationship between autophagy and senescence. Autophagy is at first reported to suppress MSC senescence by clearing injured cytoplasmic organelles and impaired macromolecules, yet recent investigations also showed that autophagy can promote MSC senescence by inducing the production of senescence-associated secretory proteins (SASP). These apparently contrary contributions of autophagy may mirror an intricate image of autophagic regulation on MSC senescence. We here tackle the pro-senescence and anti-senescence roles of autophagy in MSCs while concentrating on some possible mechanistic explanations of such an intricate liaison. Clarifying the autophagy/senescence relationship in MSCs will help the development of more effective and safer therapeutic strategies.

## Introduction

Mesenchymal stem cells (MSCs) are the most widely utilized adult stem cells in clinical trials (Trounson and McDonald, 2015). Thorough investigation has substantiated the capability of MSCs to differentiate into cells of the mesenchymal lineage including osteoblasts, chondrocytes and adipocytes, and to secrete several trophic components able to exert their effect at cellular level like apoptosis and differentiation, at systemic level like immune response modulation and at tissue level like angiogenesis and fibrosis; further, MSCs sustain cardiac, muscle, and neural tissue regeneration (da Silva Meirelles et al., 2009; Caplan and Correa, 2011). Cellular senescence and autophagy are stress responses essential for MSC homeostasis.

Senescence, biologically described as a cellular condition in which cells have lost the proliferative capacity, yet maintaining their metabolic activity, is a genetically based program responding to stress signals that avoids damaged cells to further proliferate, acting as a powerful tumor suppressive mechanism (Kuilman et al., 2010; López-Otín et al., 2013; Muñoz-Espín and Serrano, 2014; Childs et al., 2015). As reviewed elsewhere (Turinetto et al., 2016), human MSCs react with senescence induction in response to various stress stimuli, including telomere shortening (Baxter et al., 2004), oxidative stress (Stolzing and Scutt, 2006; Burova et al., 2013; Kim et al., 2011), heat shock (Alekseenko et al., 2014), and chemotherapeutic agents (Seifrtova et al., 2013; Minieri et al., 2015; Skolekova et al., 2016). Besides demonstrating that senescence program activation is independent of the MSC tissue source, those previous contributions added several details in MSC senescence profiles and phenotypes. Specifically, similar to human fibroblasts (Rodier et al., 2009), human MSC senescence program was sustained by persistent DNA damage repair activation, evidenced by the detection of characteristic enlarged nuclear foci, containing γH2AX and 53BP1 proteins (Cmielova et al., 2012; Seifrtova et al., 2013; Minieri et al., 2015; Turinetto and Giachino, 2015). At a molecular level, the overall network is not yet completely clarified, however, so further studies are required to better comprehend the mechanisms of senescence in MSCs.

Macroautophagy (hereafter referred to as autophagy and the focus of this review) represents a crucial path for the maintenance of cellular homeostasis under both physiologic and stressful situations since upon activation it sustains cellular survival thanks to the preservation of suitable metabolic functions, bioenergetic levels and amino acid pools. It is a degradation process of cellular own elements relying on the lysosomal compartment, representing a fundamental protective answer to tough situations like nutrient deprivation, where active recycling of cellular components are needed to guarantee energy homeostasis (Kang and Avery, 2008; Kroemer et al., 2010; Choi et al., 2013). In addition to working as a straightforward means to degrade large molecules that have formed aggregates or become misfolded, it is implicated in the clearance of altered and non-functional organelles including mitochondria in order to maintain appropriate cell metabolism (He and Klionsky, 2009; Boya et al., 2013). The first step of autophagy, the autophagosome biogenesis, relies on a core machinery consisting of *ATG* (autophagy-related) genes and ATG proteins; their first identification in the yeast (Tsukada and Ohsumi, 1993) was followed by cloning of their mammalian homologs, that were found to guarantee similar functions (Mizushima et al., 2011). A macromolecular complex is involved in autophagosome nucleation, consisting in the class III phosphatidylinositol 3-kinase and Beclin 1; this complex is also implicated in the phagophore membrane positioning of several other autophagic proteins that need to be subsequently recruited (Cao and Klionsky, 2007). An elongation step at the level of the phagophore membrane follows; it is performed by two ubiquitin-like systems, comprising the ATG12–ATG5-ATG16L1 complex and MAP1LC3/LC3 (microtubule associated protein 1 light chain 3). LC3 molecule is then cleaved to produce cytosolic LC3-I through the action of ATG4. LC3-I is covalently bound to phosphatidylethanolamine through the action of ATG7, ATG3 and the ATG12–ATG5-ATG16L1 complex generating LC3-II. LC3-II represents a very useful indicator of the mature autophagosome being strictly associated with the phagophore and autophagosome membrane, so it is largely employed as a marker of autophagy (Kabeya et al., 2000, 2004). Despite the fact that autophagy process was first acknowledged as a non-selective, lysosomal degradation mechanism, referred to as general autophagy, growing evidence sustains the existence of a selective autophagy, a form of autophagy mediating the degradation of specific classes of target molecules.

The intricate relationship between autophagy induced by diverse extra- or intracellular stimuli and the molecular targets that influence MSC proliferation, differentiation, and stemness has been reviewed elsewhere (Guan et al., 2013; Sbrana et al., 2016). Briefly, in human MSCs, the detection of consistent LC3-I to LC3-II conversion rates suggests constitutive activation of the autophagic flux (Oliver et al., 2012; Salemi et al., 2012) and MSC commitment to various cell lineages relies on basal autophagy activities, more than anything towards the osteoblastic lineage. Collection of undegraded autophagosomes and reduced autophagic turnover in undifferentiated MSCs have been evidenced, while in contrast stimulation of osteogenic differentiation resulted in a steady turnover increase (Nuschke et al., 2014). Conceptually, autophagy and senescence display common features as both partake in stress cell responses that can have either cytoprotective or cytotoxic consequences. However, whether autophagy performs as a positive or negative regulator of senescence in MSCs is so far debated.

## Anti-Senescence Role of Autophagy in MSCs

The main view of autophagy’s impact is an efficacious anti-senescence role implicating various pathways, whose principal players are the (mammalian) target of rapamycin (mTOR), insulin-like growth factor (IGF) binding to insulin-like growth factor receptors (IGF1R), adenosine monophosphate-activated protein kinase (AMPK) and p53 (Rubinsztein et al., 2011). Indeed, prolonged autophagy impairment in primary human fibroblasts provided through knocking down ATG7 or ATG5 resulted in cellular senescence, due to mitochondrial impairment and accumulation of produced reactive oxygen species (ROS) (Kang et al., 2011). Consistent with this view, increased autophagic activity was described to be capable of extending the lifespan of aged mice and elder flies and ripristinating the self-renewal stem cell activity, providing indications that the anti-aging effect was at least in part dependent on stem cell function restoration (Simonsen et al., 2008; Harrison et al., 2009).

More recent investigations have confirmed that autophagy is requested for sustaining the stemness and differentiation properties of stem cells. Garcia-Prat et al. described a critical role for basal autophagy in the maintenance of an immature stage in satellite cells, and unsuccessful autophagic activity promoted cell senescence defined by numerical and functional decrease of these cells (García-Prat et al., 2016).

Along this line, it was found that autophagic activity of old bone marrow-derived MSCs was diminished in comparison with young MSCs (Ma et al., 2018). Authors reported that autophagy exerted an important function in the maintenance of MSCs upon aging, and demonstrated that autophagic control could partly rescue aged MSCs’ features and bone loss in mice through the regulation of ROS-p53 (Ma et al., 2018). Those evidence suggested that the autophagic activity of MSCs could at least in part regulate bone aging, allowing to speculate the diminished autophagic activity in aged MSCs as one of the mayor sources of their degenerative modifications, and bone loss caused by impaired autophagy as an inherent novel component of bone aging.

The results of premodulated autophagy on MSC senescence were explored by up- or down-regulating autophagy through the employment of rapamycin or 3-methyladenine, respectively, prior to induction of D-galactose-mediated MSC senescence (Zhang et al., 2020). These studies exemplified that the use of rapamycin for 24 h reduced MSC senescence significantly in this experimental setting, and this was accompanied by diminished ROS production. Downregulation of p-Jun N-terminal kinases (JNK) and p-38 expression could also be demonstrated in the rapamycin treated cells (Zhang et al., 2020). In addition, the protective role of rapamycin on MSC aging could be counteracted by increasing the level of ROS, and the use of p38 inhibitors could revert the senescence induction effect of H_2_O_2_ on MSCs (Zhang et al., 2020). Altogether, this study indicated that autophagy exerted a protecting effect on D-gal-induced MSC senescence, and ROS/JNK/p38 cascade played a relevant mediating function in autophagy-mediated delay of MSC senescence. The evidence that autophagy could protect MSCs from oxidative stress signaling (Song et al., 2014) represented another indication that autophagy exerts a preserving function during cell aging. Finally, in many cases of acute senescence the autophagy flux is seriously compromised in MSCs, further evidencing that the autophagic process counterbalances damaging paths, and its negative modulation favors a senescent state (Capasso et al., 2015).

## Pro-Senescence Role of Autophagy in MSCs

Conversely, autophagy markers have been observed in senescent cells and autophagy has been demonstrated to be necessary for preservation of replicative senescence of MSCs (Zheng et al., 2014). Along this line, Zheng et al. showed that autophagy increased when MSCs entered the replicative aging state, with p53 contributing a relevant function in the autophagic increment in this specific setting (Zheng et al., 2016). p53 downregulation through knockdown experiments resulted in diminished LC3-II conversion and increased mTOR expression, thus showing that it represents a crucial trigger for autophagic activation in the course of *in vitro* expansion of MSCs (Zheng et al., 2016). Indeed, in replicative senescent MSCs, up-regulation of autophagy related genes was detected, however p53 not only played a crucial role in senescence but was also essential for triggering autophagy during culture expansion of MSCs (Fafián-Labora et al., 2019).

Further experimental models showed that the senescence status was somehow dependent on a preliminary autophagy induction. As an example, diminished insulin-like growth factor 1 (IGF1) expression was found to protect senescent MSCs kept under conditions of hypoxia by means of an up-regulated autophagic flux, thus augmenting the survival of senescent MSCs after myocardial infarction transplantation (Yang et al., 2018). Also, high glucose levels were reported to induce senescence by triggering the formation of ROS and upregulating autophagy in MSCs (Chang et al., 2015). In the above work, MSCs cultivated in high glucose concentration medium exhibited premature senescence, as showed by telomeric impact and genomic instability; it was undoubtedly evidenced that autophagy upregulation, detected through increased Beclin-1, Atg 5 and 7 expression, and augmented LC3-II conversion rate, correlated with senescence induction in MSCs while, on the opposite, negative regulation of autophagy employing 3-methyladenine prevented cellular degeneration (Chang et al., 2015).

Lastly, a few reports described oncogene-induced senescence (OIS) in MSCs in conjunction with disease manifestation. In patients suffering from systemic lupus erythematosus, for example, leptin and Neutrophil-Activating Peptide 2 sustained MSC senescence through activation of the PI3K/AKT signaling (Chen et al., 2015). Another oncogene, ASPL-TFE3, was demonstrated to induce MSC senescence through p21 up-regulation in alveolar soft part sarcoma (Ishiguro and Yoshida, 2016). In this type of induced senescence, Young et al. reported for the first time in fibroblast cells a causal association between autophagy and senescence, demonstrating that autophagy was activated during OIS and, in particular, it regulated the SASP at a post-transcriptional level, leading to the interesting speculation that autophagy provided the building blocks for the SASP protein production (Young et al., 2009). When a specialized type of general autophagy known as the TOR autophagy spatial coupling compartment or TASCC was later identified to be responsible for the protein synthesis of some SASP factors, previous speculation could be corroborated (Narita et al., 2011). The field, however, awaits further research to assess whether in MSCs similar pro-senescence autophagic activities take place during OIS.

## Reconciliation

From these previous data, it appears that autophagy acts in MSCs as either a pro-senescence or an anti-senescence process, thus if and in what manner autophagy directs MSC aging remains elusive (Table 1).

**TABLE 1:** Types of autophagy and their effect on senescence in MSCs and other cell types.

Cellular model	Type of autophagy	Effect on senescence	Senescence stimulus	References
MSC	General	Anti-senescence	Replicative exhaustion	Ma et al., 2018
MSC	General	Anti-senescence	D-galactose	Zhang et al., 2020
MSC	General	Anti-senescence	Oxidative stress	Song et al., 2014
MSC	General	Anti-senescence	Oxidative stress	Capasso et al., 2015
			Doxorubicin	
			X-ray	
			Replicative exhaustion	
MSC	General	Pro-senescence	Replicative exhaustion	Zheng et al., 2014
MSC	General	Pro-senescence	Replicative exhaustion	Zheng et al., 2016
MSC	General	Pro-senescence	Replicative exhaustion	Fafián-Labora et al., 2019
MSC	General	Pro-senescence	Hypoxia	Yang et al., 2018
MSC	General	Pro-senescence	Glucose	Chang et al., 2015
MSC	Selective	Anti-senescence	Lamin accumulation	Lee et al., 2018
MSC	Selective	Pro-senescence	Lamin accumulation	Infante et al., 2014
Fibroblast	Selective	Anti-senescence	OIS	Kang et al., 2015
Fibroblast	General (TASCC)	Pro-senescence	OIS	Narita et al., 2011
Lung primary cell	Selective	Pro-senescence	OIS	Dou et al., 2015

Although the molecular bases underpinning senescence, particularly those overlapping with autophagy, are yet poorly comprehended, reconciling these antithetic phenomena would be feasible only by speculating that autophagy may regulate a number of targets oppositely acting to modulate cellular senescence in MSCs. Interestingly, recent studies disclosed distinct functions of general versus selective autophagy in the control of senescence, partially solving seemingly conflicting evidence concerning the relation between these two fundamental homeostatic responses to stress stimuli. In model cells other than MSCs, Kwon et al. suggested the interesting possibility that such a dual role might be context and time dependent as well as specifically depend on the type of autophagy, general versus selective, involved (Kwon et al., 2017).

According to this model, under normal conditions, general autophagy would act as an anti-senescence process by preserving cellular homeostasis. Upon situations of induced stress, early action of general autophagy would also play the role of a homeostatic response, thus prevalently anti-senescent (with the exception of some specialized types of general autophagy like the TASCC). However, general autophagy exerted in cells that have already initiated a senescence process can become pro-senescent, in the sense that it can sustain viability of senescent cells. Indeed, senescent cells cannot dilute toxic byproducts as they do not undergo mitosis and they secrete a number of factors that can induce endoplasmic reticulum stress; in these conditions, autophagy induction could counteract the risk of proteostasis disruption and avoid cell death (Kwon et al., 2017). Accordingly, the anti-senescence autophagy roles reported above for MSCs were mainly related to general autophagy, and autophagy manipulation experiments were conducted before senescence induction. Conversely, the pro-senescence roles of general autophagy described in MSCs referred to either long-term cultured cells, with a presumably already initiated senescence pathway, or, possibly, to oncogene-induced senescence.

In the case of selective autophagy, when only a certain type of substrates is degraded, the resulting role on senescence would depend on the specific substrates and autophagic receptors involved. While looking for regulators of cellular senescence, Kang and Elledge (2016) identified GATA4 as a crucial regulator of the SASP and senescence. GATA4 is a transcription factor whose protein stability increases during cellular senescence leading to its accumulation. Intriguingly, authors showed a clear autophagic regulation of GATA4: under normal conditions, it is degraded thanks to the autophagic receptor protein SQSTM1/p62, yet when the cell encounters senescence-inducing stimuli, a steady decrease of the interaction between GATA4 and SQSTM1/p62 occurs, autophagic degradation is limited and GATA4 accumulates (Kang and Elledge, 2016). This accumulated GATA4 starts a transcriptional activity to switch on NFKB/NF-kB and in turn the SASP. Interestingly, GATA4-dependent regulation of the secretory phenotype was recently found to play an important role in human MSC aging (Lee et al., 2018). On the other hand, LC3B-lamin B1-dependent selective autophagy of nuclear lamina was found to act as a pro-senescence mechanism (Dou et al., 2015). Interestingly, a model of human aging based on MSCs with accumulated prelamin has been proposed (Infante et al., 2014). Taken altogether, these data seem to suggest that selective autophagy actively suppresses cellular senescence through the degradation of a senescence regulator, GATA4, whereas it promotes cellular senescence through the degradation of nuclear lamina and open the way for a similar dual control of autophagy over senescence also in MSCs.

**FIGURE 1:**
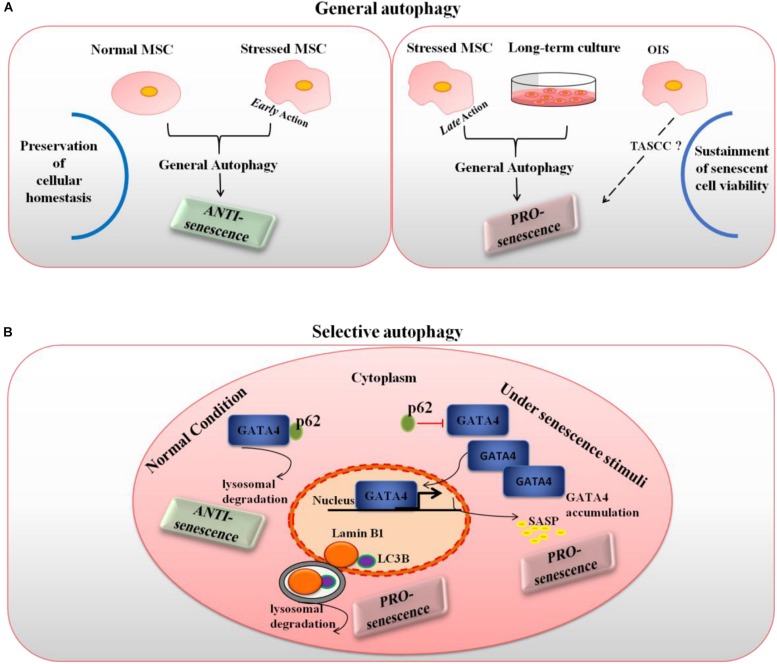
Type of autophagy, time of action and context as the frame to explain the dual role of autophagy in MSC senescence. **(A)** General autophagy, under normal conditions and when exerting early action in stressed cells, is mainly anti-senescence as it maintains cellular homeostasis; however, in late stressed and long-term cultured MSCs (pro-senescent cells) autophagy becomes pro-senescence as it can manage several senescence-associated stresses maintaining senescent cell viability; it is also possible that, under OIS, a specialized form of general autophagy known as TASCC might occur in MSCs. **(B)** Selective autophagy is pro or anti-senescence depending on the specific substrates and context involved: in normal conditions, p62-dependent autophagy specifically degrades GATA4, a main regulator of the SASP, thus actively suppressing cellular senescence, yet when the cell encounters senescence-inducing stimuli, a steady decrease of the interaction between GATA4 and p62 occurs and accumulated GATA4 transcriptionally activates the SASP; on the other hand, LC3B-lamin B1-dependent selective autophagy of nuclear lamina acts as a pro-senescence mechanism.

So at least three elements, i.e., the context, time of action and type of autophagy involved appear to constitute the frame in which autophagy can result either pro or anti-senescence in MSCs, as depicted in Figure 1. However, a few intriguing open questions concerning the extent to which cell-specific features, such as cell origin, metabolic status and age might contribute the final effect will deserve further investigation.

## Clinical Implications

When tissue homeostasis is disrupted due to MSC senescence the possible shortcoming is twofold: a loss of repairing capability caused by decreased self-renewal/differentiation abilities and a detrimental microenvironment modulation by senescent MSCs due to secretion of pro-inflammatory and matrix-degrading molecules contained in the SASP. Both have important clinical implications, so clarifying the autophagy/senescence relationship in MSCs might have an impact for the development of more effective and safer therapeutic strategies.

As an example, the regulation of autophagy in MSCs exemplifies a conceivable strategy which, influencing MSC characteristics, may hit their regenerative potential, both in terms of differentiation properties and engraftment ability (Ceccariglia et al., 2020). Activation of autophagy promoted osteogenesis in bone marrow-derived MSCs isolated from osteoporotic vertebrae (Wan et al., 2017) and prevented bone loss in elderly mice, suggesting that autophagy has a crucial role in the aging of MSCs; in this setting, autophagy upregulation could partly revert this senescence process exemplifying a likely therapeutic strategy for clinically treating age-related bone loss (Ma et al., 2018). Further, evidence indicated that modulation of autophagy in MSCs prior to their transplantation enhanced survival and viability of engrafted MSCs and promoted their pro-angiogenic and immunomodulatory characteristics (Jakovljevic et al., 2018). Some organic molecules and metabolites showed a role in autophagy/senescence modulation: cholesterol retarded senescence in bone marrow-derived MSCs by modulating autophagy (Zhang et al., 2016) while kynurenine inhibited autophagy and promoted senescence in aged bone marrow-derived MSCs through the aryl hydrocarbon receptor pathway (Kondrikov et al., 2020) which might represent a novel target to prevent or reduce age-associated bone loss and osteoporosis. Lately, to promote the efficiency of MSCs for clinical therapies, not only the intrinsic aging of these cells *in vivo* but also their aging *in vitro* upon culture expansion poses a tangible burden and should be counteracted. Among possible druggable pathways, autophagy manipulation during MSC expansion has been proposed through the employment of FDA-approved drugs like rapamycin and its derivatives (Rossi et al., 2019), which might help assessing the possibility of pharmacological extension of maximal cell lifespan while simultaneously enhancing MSC regulatory properties; further, novel small molecules known to selectively sense and react to acidic pH with high sensitivity were proved capable of promoting lysosomal acidification and inhibiting senescence in cultured MSCs through autophagy induction (Wang et al., 2018).

These and future studies on relevant targets and small molecules leading to the control and maintenance of optimal levels of autophagy might open up the way to new strategies for improving MSC transplantation.

## Author Contributions

RR and CG conceived and wrote the review. EV cooperated in bibliographic searches and table and figure editing.

## Conflict of Interest

The authors declare that the research was conducted in the absence of any commercial or financial relationships that could be construed as a potential conflict of interest.

## Abbreviations

ATG: autophagy-related
LC3: light chain 3
MSC: mesenchymal stem cell
mTOR: mammalian target of rapamycin
OIS: oncogene induced senescence
ROS: reactive oxygen species
SASP: senescence associated secretory pathway
TASCC: TOR autophagy spatial coupling compartment.
